# Household air pollution and under-five mortality in sub-Saharan Africa: an analysis of 14 demographic and health surveys

**DOI:** 10.1186/s12199-020-00902-4

**Published:** 2020-11-04

**Authors:** Fanuel Meckson Bickton, Latif Ndeketa, Grace Thandekire Sibande, Juvenal Nkeramahame, Chipiliro Payesa, Edith B. Milanzi

**Affiliations:** 1 Malawi-Liverpool-Wellcome Trust Clinical Research Programme, Blantyre, Malawi; 2 College of Medicine, University of Malawi, Blantyre, Malawi; 3 Faculty of Medicine and Health Sciences, University of Antwerp, Antwerp, Belgium; 4 Medecins Sans Frontieres/Epicentre Mbarara Research Center, Mbarara, Uganda; 5 MRC Clinical Trials Unit, University College London, 90 High Holborn, WC16LJ, London, UK

**Keywords:** Household air pollution, Under-five mortality, Sub-Saharan Africa, Demographic Health Survey

## Abstract

**Background:**

Globally, over four million deaths are attributed to exposure to household air pollution (HAP) annually. Evidence of the association between exposure to HAP and under-five mortality in sub-Saharan Africa (SSA) is insufficient. We assessed the association between exposure to HAP and under-five mortality risk in 14 SSA countries.

**Methods:**

We pooled Demographic and Health Survey (DHS) data from 14 SSA countries (*N* = 164376) collected between 2015 and 2018. We defined exposure to HAP as the use of biomass fuel for cooking in the household. Under-five mortality was defined as deaths before age five. Data were analyzed using mixed effects logistic regression models.

**Results:**

Of the study population, 73% were exposed to HAP and under-five mortality was observed in 5%. HAP exposure was associated with under-five mortality, adjusted odds ratio (OR) 1.33 (95% confidence interval (CI) [1.03–1.71]). Children from households who cooked inside the home had higher risk of under-five mortality compared to households that cooked in separate buildings [0.85 (0.73–0.98)] or outside [0.75 (0.64–0.87)]. Lower risk of under-five mortality was also observed in breastfed children [0.09 (0.05-0.18)] compared to non-breastfed children.

**Conclusions:**

HAP exposure may be associated with an increased risk of under-five mortality in sub-Saharan Africa. More carefully designed longitudinal studies are required to contribute to these findings. In addition, awareness campaigns on the effects of HAP exposure and interventions to reduce the use of biomass fuels are required in SSA.

## Background

Exposure to household air pollution (HAP) is a major public health hazard worldwide and among the top leading risk factors for global disability and mortality [1–3]. Close to four million deaths are attributed to HAP exposure annually [4]. Household use of biomass fuels is one of the largest sources of HAP globally [5]. Biomass fuels refer to fuel that comes from plant-based or animal-based material, including charcoal, wood, dung, and crop residues. The inefficient and incomplete combustion of these energy sources produces harmful smoke, including pollutants such as carbon monoxide and particulate matter–a mixture of organic and inorganic particles [6]. The use of biomass fuels for cooking substantially contributes to ambient air pollution which was attributed to five percent of all-cause mortality in 2017 [2].

The main burden of HAP is found in low-resource settings, where many people are still at the bottom of the energy ladder [5]. Women and children are disproportionately exposed in and around the home due to gender-based domestic roles associated with time spent indoors [7]. Studies indicate that under-five children are the most vulnerable group to pollutant cooking fuels with negative health consequences [8]. Exposure to HAP has been shown to be associated with intrauterine growth restriction, preterm birth, and low birth weight [9–11]. Under-five children who are exposed to HAP may also be at an increased risk for lower and acute respiratory infections (ARIs) and pneumonia [12].

The association between HAP and under-five mortality has previously been investigated by several studies. Exposure to HAP was associated with an increased risk for neonatal, child and under-five mortality in 47 countries using Demographic and Health Surveys (DHS) and among the five regions studied (America, Asia, Europe, Northern Africa, and sub-Saharan Africa), sub-Saharan Africa had the highest association between HAP exposure and childhood mortality [13]. A cross-sectional study of 23 countries in SSA by Owili et al. [8], that also used DHS data, similarly found an association between the use of biomass cooking fuels and increased risk of under-five mortality. These findings are in line with studies that have been conducted outside SSA, including India and Pakistan [14, 15].

Since these studies were conducted, more recent data have been collected from the DHS since 2015 which were not included in the previous multi-country analyses, including data from South Africa whose current dataset is the first in 22 years. There is compelling evidence of an association between air pollution and infant mortality but most studies to date have focused on acute exposure and ambient air pollution mainly in developed countries [2]. This makes the research on the association between HAP exposure and under-five mortality particularly in Africa an urgent public health concern and vital to inform localized and contextual policy decisions on reducing the burden of HAP.

Therefore, we set out to assess the association between exposure to HAP (i.e., biomass fuel for cooking) and under-five mortality using the most recent DHS datasets from 14 countries in SSA.

## Methods

### Study population and data collection

This study is a secondary analysis of data from DHS conducted in SSA. The details of these surveys are explained elsewhere (https://dhsprogram.com/) but, briefly, DHS are cross-sectional nationally representative household surveys that provide data for a wide range of monitoring and impact evaluation indicators in the areas of population, health, and nutrition. The samples are based on a two-stage cluster design where, firstly, Enumeration Areas (EA) are drawn from census files and, in the second stage, in each EA selected, a sample of households is drawn from an updated list of households [https://dhsprogram.com/What-We-Do/Methodology.cfm]. We used data from 14 surveys conducted between 2015 and 2018 in sub-Saharan Africa. The following countries were included in the analysis; Angola (2015), Benin (2016), Burundi (2015), Ethiopia (2016), Guinea (2018), Mali (2018), Malawi (2015), Nigeria (2018), South Africa (2016), Tanzania (2015), Uganda (2015), Rwanda (2015), Zambia (2018), and Zimbabwe (2015). Data was collected from all eligible women aged 15–49 years and analyses were limited to children born in the last 5 years prior to the survey to minimize exposure misclassification and influence of recall bias. In addition, we excluded all observations with missing cooking fuel type, or missing outcome data and twin births. The final sample size of children included in the analysis was 164376 out of 73772 households.

### Ethical considerations

Ethical clearance for the DHS in all countries was obtained from the Inner City Fund (ICF) International Institutional Review Board (IRB). Additionally, country-specific DHS protocols had been reviewed by the ICF IRB and by an IRB in the host country. As the de-identified data for the current study came from secondary sources whose data is publicly available, ethics approval for this study was not required.

### Exposure assessment

We defined the exposure of interest ‘HAP’ as the use of biomass fuels for cooking. During the DHS interviews, mothers were asked the question “What type of cooking fuel do you use?” We used responses to this question to assess their exposure to HAP. We categorized responses of wood, charcoal, dung, kerosene, crop residues, shrubs, and coal as exposed categories and natural gas, biogas, liquefied petroleum gas (LPG) and electricity as clean fuels. We used clean fuels as reference categories. We created two separate variables for HAP exposure; a binary variable where use of biomass fuels i.e. wood, charcoal, dung, crop residues, shrubs, coal, and kerosene was coded as ‘1’ and use of natural gas, biogas, LPG and electricity was coded as ‘0’ and a multinomial variable where charcoal was categorized as a separate category from other biomass fuels (coded as ‘2’) because it has been suggested as a cleaner fuel than wood [16].

### Health outcome assessment

During the interviews, mothers were also asked if their child had died before her or his fifth birthday, 5 years prior to the survey. Therefore, the outcome of this study was under-five mortality which we defined as the death between birth and the fifth birthday of the child (i.e., 0–59 months).

### Potential confounders

We adjusted for the following potential confounders: sex of the child (male or female), birth order, number of under-five children in the household, and mother’s age at birth. This is because it has been shown that children who are born within 15 months of a preceding birth are more likely than other children to die in the first 2 years of life [17] and that firstborns and advanced maternal age at birth are all associated with poor birth outcomes including under-five mortality [18, 19]. Birth order, number of under-five children, and mother’s age at birth were entered as continuous variables. Mother’s education level (none, primary, secondary, higher), occupation, residence (urban/rural) and wealth index were adjusted for as indicators of socio-economic status. The wealth index was calculated by DHS using household items (ownership of bicycles, etc.), where scores were calculated using principal component analysis and the resultant scores divided into quintiles of poorest, poorer, middle, rich, richer, and richest. Mother’s smoking status (yes/no), frequency of any household smoking (never, less than once a month, daily) was also adjusted for because maternal active and household second hand smoke exposure have also been associated with under-five mortality [8, 18, 20]. We also adjusted for kitchen location and breastfeeding status; kitchen location has been shown to influence indoor HAP exposure levels, while breastfeeding status is associated with the protective effect against infections and, therefore, may attenuate the risk of under-five mortality associated with HAP [15, 21]. We adjusted for the country (Angola arbitrarily chosen as a reference category) and year of the survey to consider the differences across the countries and time the surveys were conducted. Birth weight was not included as a confounder because it is likely on the causal pathway between exposure to HAP and mortality, and therefore adjustment of such an intermediate variable would produce biased estimates. Controlling for a factor that is on the causal pathway leads to underestimation of the strength of the effect of the exposure on the outcome under study [22, 23].

### Statistical analysis

We used mixed fixed effects logistic regression models to analyze the data with random intercepts specified for each country. We first fitted a crude model without adjustment (country was included in the crude model to obtain crude estimates per country). In adjusted models, we fitted two separate models. In model I, we modeled HAP exposure as a binary variable and, in model II, we used the multinomial exposure variable. We adjusted both models I and II for the previously mentioned confounders. Up to 93, 465 observations did not have information on either kitchen location, or frequency of smoking in the household. Therefore, we used the missing indicator method by creating an indicator variable for those with missing data on kitchen location and smoking frequency in the household.

### Sensitivity analyses

We stratified the analyses by breastfeeding status and kitchen location because of the strong evidence of protective effects of breastfeeding on child mortality and influence of kitchen location on associations of HAP exposure and health effects [21, 24]. We also explored the robustness of the estimates using the leave-one-out method to assess which countries heavily influenced the overall estimates by recalculating the results N-1 times, each time leaving out one country. We also analyzed data without replacing missing observations with indicator variables.

We performed all statistical analyses using STATA version 15 (TX, USA), and we took into account the complex survey design of the data in the survey framework. An alpha of 0.05 was considered as the level of significance.

## Results

### Study population characteristics

**Fig. 1:**
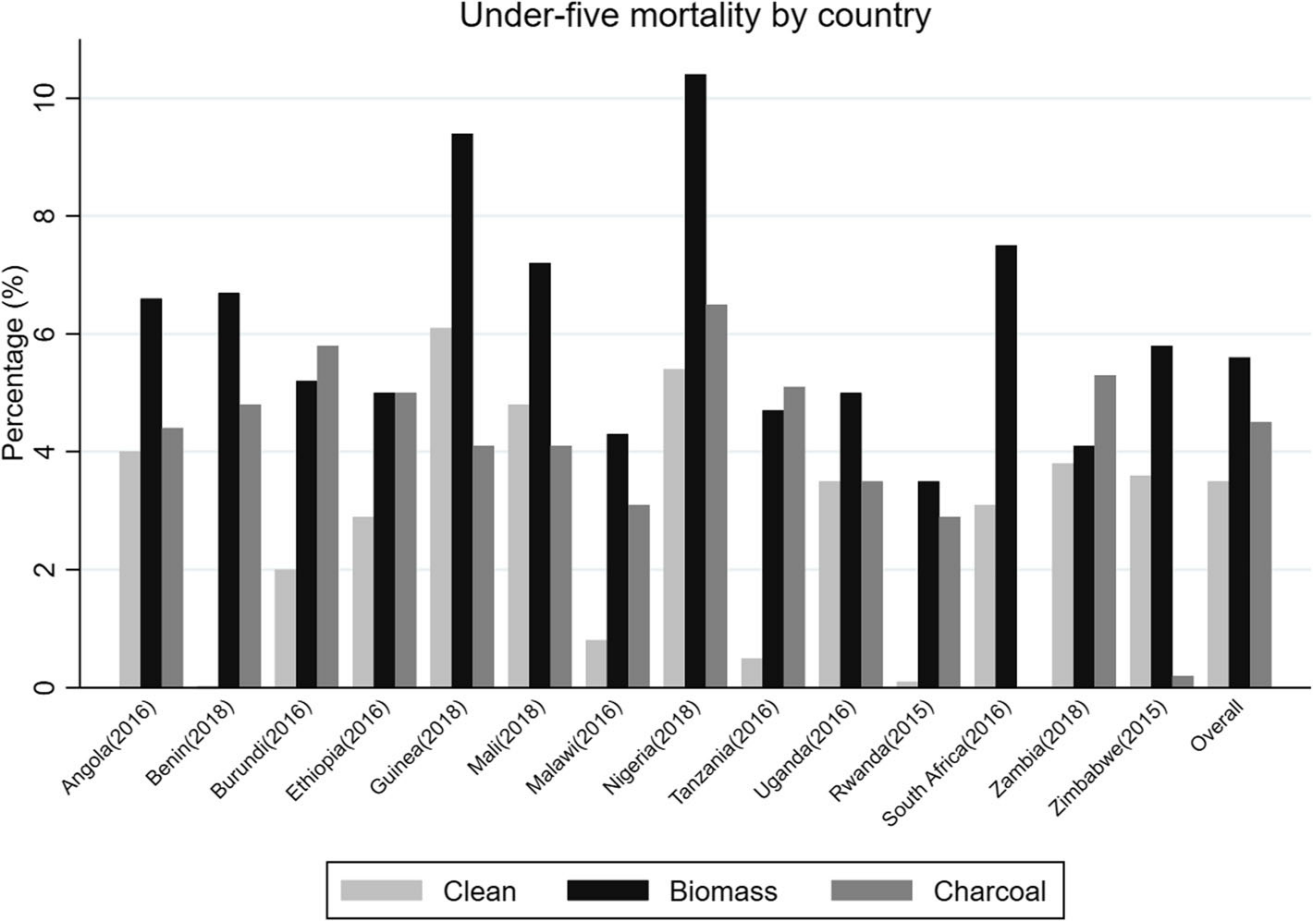
Percentage of under-five mortality by country

Table 1 shows the study population characteristics and by cooking fuel type. Other forms of biomass were the most used form of cooking fuel (72.1%) compared to charcoal (16%) and clean fuel (11.9%). At least 70.3% of the population were from rural areas and only 3.5% of the mothers had attained education higher than secondary school education level. High prevalence of HAP exposure due to biomass was observed in Ethiopia (92.1%) followed by Burundi (87.4%) and Rwanda (84.4%), but low in Angola (35.0%) and Zambia (55.5%) and 22.8% in South Africa. Prevalence of charcoal use is also listed in Table 1, and there was no reported charcoal use in South Africa. There were significant differences in mother’s education level, residence, and mother’s occupation (*p* < 0.001) among the different categories of cooking fuel. Under-five mortality proportions per country are presented in Fig. 1. The overall under-five mortality was 5.2% and 3.5%, 5.6% and 4.5% for clean fuel, other biomass, and charcoal use, respectively. Higher proportions of under-five mortality were observed in Nigeria (10.4%) and Guinea (9.4%) and the lowest in Rwanda (3.5%), Zambia (4.1%), and Malawi (4.3%).

**Table 1:** Study population characteristics by cooking fuel type (*N* = 164376)

	Total (*N* (%)) *N* = 164376	Clean fuel (%) *N* = 13794	Biomass (%) *N* = 124020	Charcoal (%) *N* = 26562	*p* value
Fuel use (total)		11.9	72.1	16.0	< 0.001
*Country (year)*^*α*^					
Angola	13706 (8.3)	5391 (48.9)	5226 (35.0)	3089 (16.0)	
Benin	12632 (7.6)	396 (2.82)	9337 (74.4)	2899 (22.7)	
Burundi	12728 (7.7)	20 (0.1)	10784 (87.4)	1924 (12.3)	
Ethiopia	10119 (6.1)	508 (3.2)	8705 (92.1)	906 (4.7)	
Guinea	7495 (4.5)	96 (1.4)	5334 (70.2)	2065 (28.4)	
Mali	9496 (5.7)	134 (0.7)	7468 (82.4 )	1894 (16.8)	
Malawi	16486 (10.0)	186 (1.5)	13809 (83.8)	2491 (14.6)	
Nigeria	32133 (19.5)	2384 (9.1)	28026 (84.7)	1723 (6.0)	
Tanzania	9269 (5.6)	30 (0.4)	7228 (75.5)	2011 (24.1)	
Uganda	14498 (8.8)	40 (0.3)	11368 (76.3)	3090 (23.3)	
Rwanda	7469 (4.5)	14 (0.1)	6184 (84.4 )	1271(15.5)	
South Africa^β^	3289 (2.0)	2392 (77.1)	897 (22.8)	-	
Zambia	9399 (5.7)	518 (6.5)	5690 (55.5)	3191 (37.9)	
Zimbabwe	5657 (3.4)	1685 (24.7)	3964 (75.1)	8 (0.2)	
Child dead	9478 (5.2)	529 (8.3)	7746 (77.4)	1203 (13.7)	< 0.001
*Sex of child*					0.799
Male	83228 (50.7)	7023 (12.7)	62716 (71.3)	13489 (15.9)	
Female	81148 (49.3)	6771 (12.5)	61304 (71.5)	13073 (15.9)	
*Residence*					< 0.001
Urban	47799 (29.6)	11467 (35.4)	18283 (27.7)	18049 (36.8)	
Rural	116577 (70.3)	2327 (3.0)	105737 (89.8)	8513 (7.1)	
*Mother education*					< 0.001
None	61018 (34.2)	1092 (1.9)	53364 (86.9)	6562 (11.1)	
Primary	60357 (37.3)	2718 (5.3)	48286 (79.3)	9353 (15.3)	
Secondary	37112 (24.9)	7549 (32.8)	20602 (45.5)	8961 (21.6)	
Higher	5889 (3.5)	2435 (52.9)	1768 (19.3)	1686 (27.7)	
Age at first birth (mean (SD))	19.4 (0.1)	20.6 (0.1)	19.0 (0.02)	19.8 (0.05)	0.706
Under-five children in household (mean(SD))	1.9 (0.08)	1.7 (0.01)	2.0 (0.01)	1.7 (0.01)	< 0.001
*Mother occupation*					< 0.001
None	40055 (30.8)	4809 (44.9)	26784 (28.0)	8462 (32.6)	
Professional	6295 (3.5)	1631 (10.5)	2504 (1.4)	2160 (7.2)	
Clerical/sales	30817 16.0)	3854 (21.7)	20125 (13.2)	6838 (24.4)	
Agriculture	54207 (35.4)	584 (2.3)	50082 (46.3)	3541 (12.0)	
Services	9710 (6.6)	1627 (13.9)	5361 (4.4)	2722 (11.1)	
Manual	12487 (7.5)	762 (6.6)	8586 (6.5)	3139 (12.4)	
*Wealth index*					< 0.001
Poorest	41293 (23.2)	212 (1.5)	40520 (97.4)	561 (1.1)	
Poorer	36916 (21.9)	927 (6.2)	32924 (87.4)	3065 (6.3)	
Middle	33695 (20.0)	2900 (12.4)	26832 (77.7)	3963 (9.7)	
Richer	30381 (18.8)	3565 (17.3)	19784 (58.2)	7032 (24.4)	
Richest	27034 (15.9)	6320 (27.7)	7682 (22.9)	13032 (49.3)	
*Mother smokes*					< 0.001
Yes	1265 (0.8)	160 (22.0)	922 (63.9)	182 (14.1)	
No	161465 (99.2)	12414 (10.1)	122673 (73.3)	26378 (16.5)	
*Smoker in household*					< 0.001
Never	64687 (37.4)	6654 (14.6)	44432 (65.1)	13601 (20.2)	
Daily	7247 (4.5)	495 (14.7)	5507 (69.7)	1245 (15.5)	
<1/month	3920 (2.5)	278 (9.1)	2972 (74.1)	715 (16.7)	
Missing	93465 (55.4)	6497 (9.9)	74876 (76.9)	12092 (13.1)	
*Kitchen location*					< 0.001
In the house	22547 (15.7)	6550 (37.7)	11225 (44.7)	4772 (17.5)	
Separate building	32340 (18.5)	712 (2.2)	27629 (86.4)	3999 (11.1)	
Outdoors	23370 (13.4)	242 (0.9)	15775 (66.1)	7353 (33.0)	
Missing	91062 (53.7)	6420 (10.5)	73113 (76.7)	11520 (12.8)	
*Breastfeeding status*					< 0.001
Yes	157027 (94.7)	11461 (8.5)	119857 (74.7)	25709 (16.8)	
No	7028 (5.2)	863 (24.8)	4966 (61.2)	1199 (13.9)	

**α** weighted percentages for fuel use per country calculated out of total *N* for each country. All other weighted percentages calculated out of total per variable

### Association between HAP exposure and under-five mortality

**Fig. 2:**
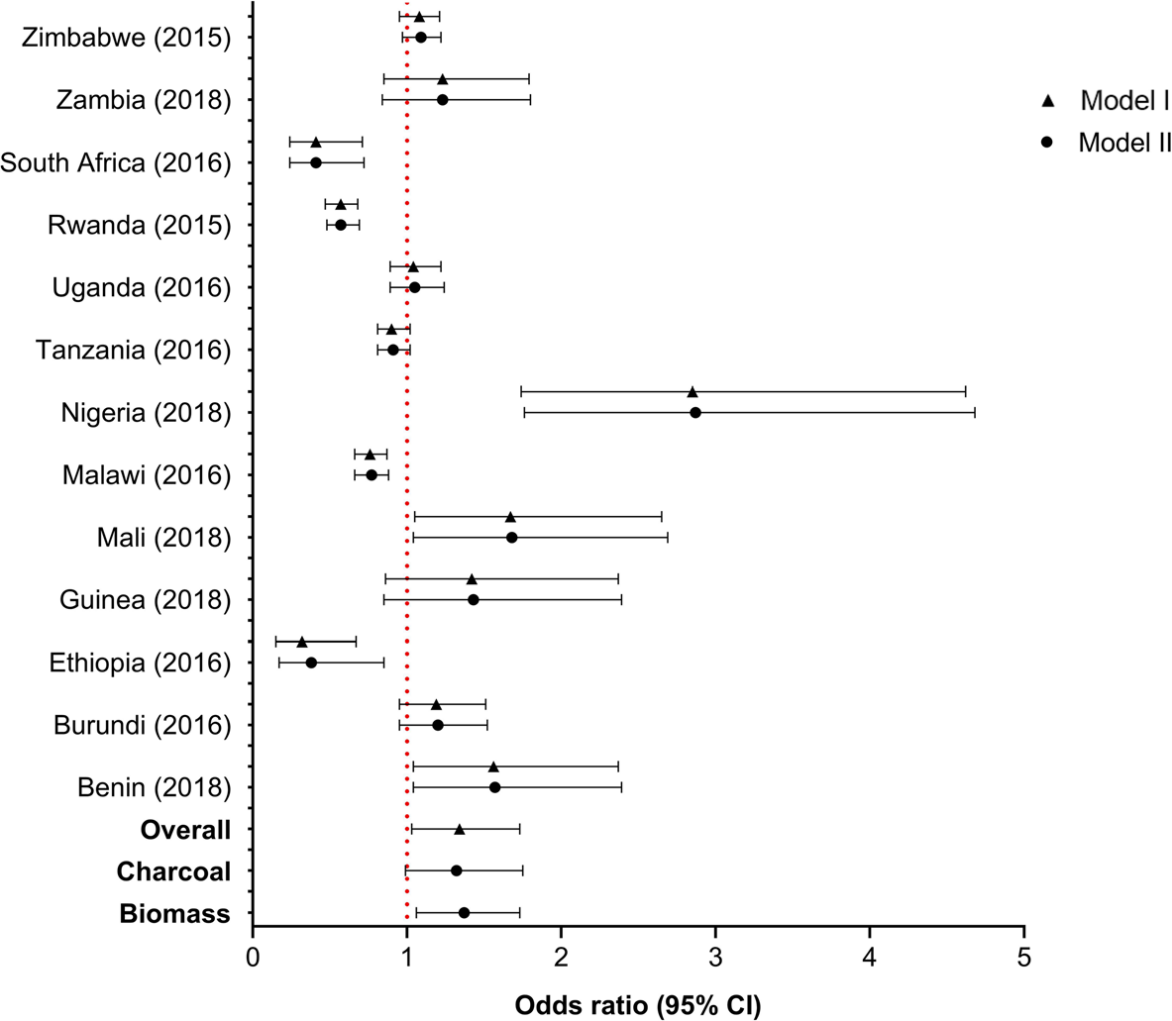
Association between HAP exposure and under-five mortality. Angola used as reference country

Fig. 2 presents the associations of HAP exposure with under-five mortality. In model I, we observed associations between HAP exposure and the risk of under-five mortality, OR 95% CI [1.33 (1.03–1.71)]. The estimates were similar when we separated charcoal from other biomass fuels, i.e., [1.35 (1.07–1.71)] for other biomass use and [1.32 (1.00–1.74)] for charcoal use only. In all adjusted models, using Angola as a reference country, associations with under-five mortality were stronger in west African countries than southern or eastern African countries, e.g., Benin [1.56 (1.04–2.37)], Mali [1.67 (1.05–2.65)], and Nigeria [2.85 (1.74–4.62)] .

Female children were at a lower risk for under-five mortality compared to male children [0.82(0.78–0.86)] (Table 2). Children born to mothers with at least secondary school [0.69 (0.57–0.84)] or higher education [0.55 (0.43–0.70)] were less likely to die before the age of five compared to children born from mothers who never had any education. Likewise, children from the upper quintiles of the wealth index (middle, richer and richest) were less likely to die than children from the poorest households. Maternal smoking and any smoking in the household by other members of the household was not associated with under-five mortality. Children from mothers who were employed had a higher risk of under-five mortality compared to unemployed mothers (Table 2).

**Table 2:** Association of HAP exposure and other risk factors with under-five mortality

	Model I		Model II	
	Odds ratios	95% CI	Odds ratios	95% CI
*Fuel*				
Clean	Ref		Ref	
Biomass fuel	1.33	1.03–1.71	1.35	1.07–1.71
Charcoal			1.32	1.00–1.74
Year	0.90	0.80–1.03	0.90	0.80–1.03
*Sex*				
Male	Ref		Ref	
Female	0.82	0.78–0.86	0.82	0.78–0.86
*Wealth quintile*				
Poorest	Ref		Ref	
Poorer	0.95	0.87–1.04	0.95	0.87–1.05
Middle	0.81	0.73–0.89	0.81	0.73–0.91
Richer	0.74	0.64–0.86	0.74	0.64–0.87
Richest	0.58	0.51–0.67	0.59	0.50–0.69
*Residence*				
Urban	Ref			
Rural	1.03	0.88–1.21	1.03	0.88–1.20
Birth order	1.05	1.03–1.07	1.05	1.03–1.07
Under five children in HH	0.38	0.30–0.49	0.38	0.30–0.49
Maternal age at birth	0.99	0.98–1.00	0.99	0.98–1.00
*Mother’s education*				
None	Ref		Ref	
Primary	0.91	0.78–1.07	0.91	0.78–1.07
Secondary	0.69	0.57–0.84	0.69	0.57–0.84
Higher	0.55	0.43–0.70	0.55	0.43–0.70
*Mother’s occupation*				
Unemployed	Ref		Ref	
Professional	0.97	0.80–1.18	0.97	0.80–1.18
Clerical/sales	1.16	1.04–1.29	1.16	1.04–1.30
Agriculture	1.12	1.03–1.20	1.11	1.03–1.20
Services	1.31	1.07–1.59	1.31	1.07–1.59
Manual	1.04	0.92–1.18	1.04	0.92–1.18
Maternal smoking	1.15	0.82–1.62	1.15	0.82–1.62
Breastfed	0.09	0.05–0.18	0.09	0.05–0.18
*Kitchen location*				
Inside	Ref		Ref	
Separate building	0.85	0.73–0.98	0.84	0.74–0.97
Outside	0.75	0.64–0.87	0.75	0.64–0.87
*Smoking in the household*				
Never	Ref			
Daily	1.10	0.90–1.35	1.10	0.90–1.35
< 1/month	1.14	0.91–1.43	1.14	0.91–1.43

### Sensitivity analysis

**Fig. 3:**
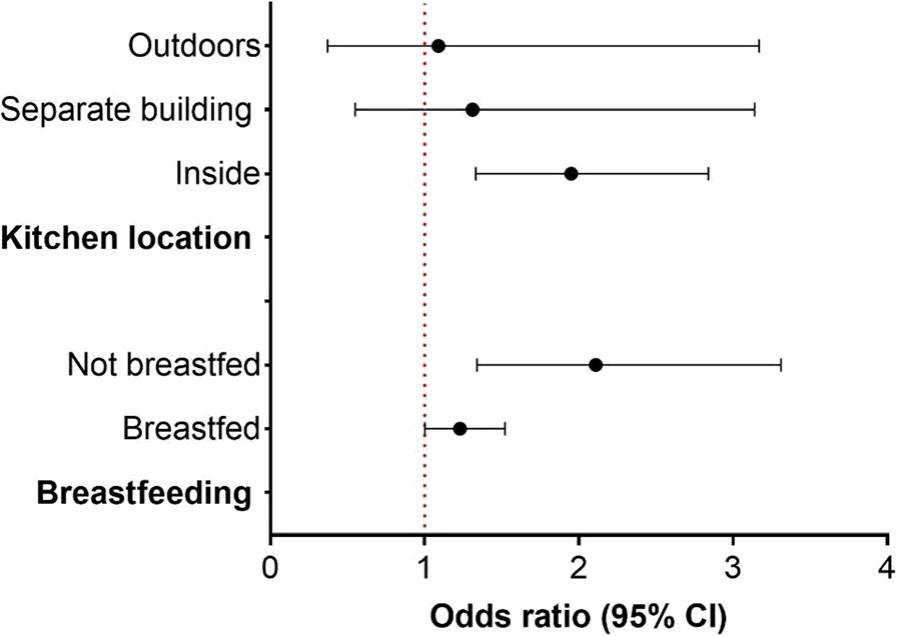
Stratified analyses by kitchen location and breastfeeding status

**Fig. 4:**
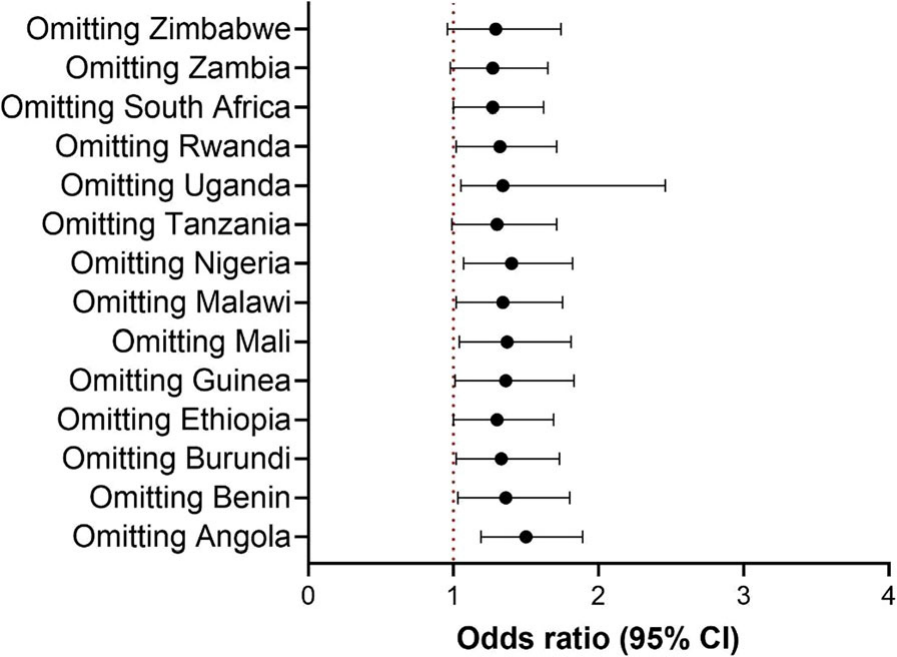
Association between HAP exposure and under-5 mortality: leave-one-out analysis for model I

Figure. 3 presents the association between HAP and under-five mortality stratified by kitchen location and breastfeeding status. We conducted all sensitivity analyses using the binary response variable. Children who lived in a house with an in-house kitchen and used biomass for cooking had increased risk of under-five mortality, [1.95 (1.33–2.95)]. There was also a suggested higher risk for those with a kitchen in a separate building [1.31 (0.55–3.14] or outdoor kitchen [1.09 (0.37–3.17)] compared to households with kitchen inside, but this was not significant (interaction *p* value = 0.684). While we observed an increased risk for both breastfed and non-breastfed children, associations of HAP exposure with under-five mortality were stronger in non-breastfed children [2.11 (1.34–3.31)] (interaction *p* value = 0.004). Leave-one-out analyses also showed consistent results as in the main analyses (Fig. 4) and analyses without using the missing indicator variable methods also showed similar results (Additional file 1: Table S1).

## Discussion

In this pooled multi-country analysis of DHS from SSA between 2015 and 2018, HAP exposure was associated with risk of under-five mortality, and this was consistent regardless of kitchen location and breastfeeding status.

Our findings concur with previous SSA multi-country analyses on the associations of HAP exposure and under-five mortality [8, 13, 25], thereby contributing to and strengthening the limited evidence on the association between HAP exposure and under-five mortality in Africa. It is not surprising that these associations have not been observed in high-income countries as the prevalence of biomass use is very low in those settings as compared to SSA, a largely low-income setting [13, 26]. It, therefore, follows that the evidence on the effects of air pollution in developed countries is largely in the context of outdoor air pollution. In SSA, higher mortality is attributed to HAP than outdoor air pollution, although this could be due to the dearth of literature on the effects of outdoor air pollution in the region. It is reported that deaths in Africa from outdoor air pollution have increased from 164000 in 1990 to 258000 in 2017—a growth of nearly 60%, highlighting the urgent need to tackle this problem too in Africa [1].

We found lower risk of mortality for female children than male children, in line with previous studies [8, 15]. Biologically, boys are considered to be at higher risk of respiratory related adverse health effects than girls because of their lower respiratory volumes and narrower peripheral airways in infancy [27] which may contribute to their increased vulnerability to under-five mortality. There was also reduced risk of mortality in children from richer households. This was also reflected in the association observed with the mother’s education, reflecting the influence of socio-economic status on the relationship between HAP and under-five mortality. Mothers with higher socio-economic status are more likely to live in more conducive conditions that provide favorable environment for greater health indicators such as better nutrition and access to healthcare which are very crucial in the first 5 years of life. In contrast, low-income households, with poor quality housing and poverty, are more likely to rely on polluting energy sources for their cooking which compounds the health risks associated with their use [28]. Although not significant, there was a suggested high risk of mortality in children whose mothers or household members smoked inside the household. The prevalence of second-hand smoke exposure in our study was small (4.5%), but these findings might suggest that exposure to second-hand smoke can possibly aggravate associations of HAP with mortality. Owili et al. [29] investigated the association between second-hand smoke exposure and under-five mortality and found stronger positive associations with under-five mortality.

In sensitivity analyses, we consistently observed an increased risk of under-five mortality in both children that were breastfed and those that were not breastfed in contrast with other studies [8] and despite evidence of the protective effect of breastfeeding on other adverse health outcomes in children in relation to air pollution exposure [30]. However, associations were stronger in children whose mothers did not breastfeed and associations for the children that were breastfed were only borderline significant, therefore partly aligning with previous reports [15].

There was higher risk of under-five mortality in children from employed mothers. This was an unexpected finding and in contrast with studies that have shown lower risk of under-five mortality in children with employed mothers [31]. However, our finding is in line with other studies where higher risk of under-five mortality was observed in employed mothers [32]. The risk of under-five mortality in our study was higher among children of mothers employed in clerical/sales, agriculture, and manual work—all of which are mostly informal—compared to those in professional (formal) employment. In Nigeria, clerical/sales, agriculture, and manual work were all found to have the poorest coverage of childhood vaccination [33]. The increased risk of under-five mortality among working mothers could be a result of inadequate attention to childcare; lack of personal and timely care, including infrequent breast feeding [32].

The mechanisms by which the by-products released from HAP operate are multidimensional and complex. The large quantities of particulate matter and other pollutants emitted from use of biomass fuels for cooking such as suspended particulate matter, nitrogen dioxide, polycyclic aromatic hydrocarbons are all known to be toxic and carcinogenic [34]. Children are especially vulnerable during foetal development and in their earliest years, while their lungs, organs, and brains are still maturing. They breathe faster than adults, taking in more air and, with it, more pollutants [28]. In addition, particulate matter is suggested to cause inflammation of pathways in the lungs resulting in respiratory complications such acute respiratory infections in children [35] which is one of the leading causes of deaths of infants and under-five children in SSA [36–38]. Furthermore, HAP exposure in utero exposes infants to risk of constricted fetus growth, resulting in low birthweight, all of which can lead to death in infants and very young children [10].

We analyzed all-cause mortality in our study because DHS does not collect cause-specific mortality data. In the African region, acute respiratory infection is the leading cause of death of under-five children and premature birth is the only factor that kills more under-five children globally than acute respiratory infections [28]. The association between HAP from cooking fuel and ARI in Africa has been strongly suggested [12] as such, deaths observed in this analysis may be largely because of ARI. However, in 2018, the World Health Organization (WHO) estimated that in under-five children, at least 184 deaths per 100,000 were attributed to HAP in low- and middle-income countries (LMICs), the majority of which are in SSA, but this decreased dramatically to 12.9 in children aged 5–14 years [28].

While studies of improved stove interventions to reduce HAP in LMICs, including SSA, have shown that improved stoves can reduce exposure to HAP resulting from solid fuel smoke [39], which may in turn reduce the risk of under-five mortality, other intervention studies have failed to demonstrate the benefit of improved stoves in other health outcomes in children [40]. This may be because Africa has a significantly lower rate of access to clean and improved stoves than any other region globally, e.g, as of 2014, only 5% of Africans used “clean” cookstoves that run on fuels, such as liquefied petroleum gas (LPG) (5%) and a growing number of SSA households (about 3.5%) used intermediate improved cookstoves which are substantially more fuel efficient but do not achieve the emission reductions needed to realize the full health and environmental benefits of clean cooking [41]. In addition, within countries, rural households seem to be less aware and capable of affording clean cooking stoves compared to urban consumers [42]. This highlights the need for more carefully designed longitudinal studies focusing on African populations to understand the relationship between HAP exposure and under-five mortality and the role of interventions in reducing HAP exposure.

While our study has the strength of national representative data collected in a systematic and validated manner, and that we also used the most recent datasets that were not used in previous multi-country analyses, our findings need to be interpreted in the context of some limitations. Firstly, we used cross-sectional secondary datasets to assess associations of HAP exposure with under-five mortality. This presents several potential sources of bias such as misclassification of exposure where households may use more than one type of cooking fuel, besides the primary cooking fuel type. However, we expect this misclassification of exposure to be non-differential. We also relied on self-reported responses regarding information of the children which might increase the possibility of recall bias. We were also unable to adjust for the possibility of cooking on open fire or closed fires as this variable is not available for most recent datasets in the DHS, and this may bias our results as cooking on open or closed fires may influence levels of exposure [43]. Lastly, due to the cross-sectional nature of the study, we were not able to establish a temporal link between HAP exposure and under-five mortality.

## Conclusions

In conclusion, cooking using biomass fuels, which causes HAP exposure, is a modifiable risk factor that is associated with under-five mortality in SSA populations. More carefully designed longitudinal cohort studies, awareness campaigns or programs on the effects of HAP exposure, and more research on interventions to mitigate the burden of HAP exposure, including under-five mortality, are required in SSA.

## Supplementary information

**Additional file 1: Table S1.** Associations of HAP exposure and under-five mortality without missing indicator method (Model I).

## Data Availability

The study used, with permission, data from the Inner City Fund (ICF) and the Demographic Health Surveys (DHS) program. The data is publicly available upon request from the ICF on (https://dhsprogram.com/data/available-datasets.cfm).

## Abbreviations

ARI: Acute respiratory infection
CI: Confidence interval
DHS: Demographic and Health Survey
EA: Enumeration area
HAP: Household air pollution
ICF: Inner City Fund
IRB: Institutional Review Board
LPG: Liquefied petroleum gas
LMIC: Low- and middle-income country
OR: Odds ratio
SSA: Sub-Saharan Africa
WHO: World Health Organization
