# Supplementary material for: Household air pollution and under-five mortality in sub-Saharan Africa: an analysis of 14 demographic and health surveys
Source: Environ Health Prev Med. 2020 Nov 4;25:67. doi: 10.1186/s12199-020-00902-4 (PMC7643379; doi:10.1186/s12199-020-00902-4)
Supplement: Supplementary file 1 — Additional file 1: Table S1. Associations of HAP exposure and under-five mortality without missing indicator method (Model I). [file 12199_2020_902_MOESM1_ESM.docx]

**Additional file 1**

**Fanuel Meckson Bickton, Latif Ndeketa, Grace Thandekire Sibande, Juvenal Nkeramahame, Chipiliro Payesa, Edith B. Milanzi**

Table S1 Associations of HAP exposure and under-five mortality without missing indicator method (Model I)

|  |  |
| --- | --- |
| Variables | Odds Ratio (95% CI) |
|  |  |
| Clean fuels  Biomass fuel | Ref  1.54 (1.09 – 2.19) |
|  |  |
| *Country* |  |
| Angola | Ref |
| Benin | 1.20 (0.81 – 1.78) |
| Burundi | 1.12 (0.95-1.33) |
| Ethiopia | 0.24 (0.09 – 0.66) |
| Guinea | 1.13 (0.69 -1.89) |
| Mali | 1.47 (0.98-2.20) |
| Malawi | 0.64 (0.59 -0.71 |
| Nigeria | 1.95 (1.26 -3.03) |
| Tanzania | 0.69 (0.62 – 0.77) |
| Uganda | 0.85 (0.77 -0.96) |
| Rwanda | 0.44 (0.38 – 0.53) |
| South Africa | 0.22 90.14 – 0.34) |
| Zambia | 1.09 (0.76 -1.60) |
| Zimbabwe | 0.83 (0.73 – 0.96) |
| year | 0.88 (0.77- 1.01) |
| *Sex*  Male  Female | Ref  0.83 (0.78 – 0.88) |
| *Wealth quintile* |  |
| Poorest |  |
| Poorer | 0.87 (0.78 – 0.98) |
| Middle | 0.86 (0.80 – 0.94) |
| Richer | 0.72 (0.62 – 0.85 ) |
| Richest | 0.58 (0.49 – 0.70) |
| *Residence*  Urban  Rural | 1.00  1.13 (0.94 – 1.38) |
| Birth order | 1.06 (1.04 – 1.09) |
| Under five children in HH | 0.22 (0.18 – 0.27) |
| Maternal age at birth | 0.99 (0.97 – 1.02) |
| *Mother's education* |  |
| None | Ref |
| Primary | 0.92 (0.78 – 1.10) |
| Secondary | 0.76 (0.62 – 0.95) |
| Higher | 0.64 (0.41 – 1.00_ |
| *Mother’s occupation* |  |
| Unemployed | Ref |
| Professional | 1.00 (0.77- 1.30) |
| Clerical/sales | 1.16 (0.97- 1.39) |
| Agriculture | 1.07 (0.98 – 1.18) |
| Services | 1.32 (1.01 – 1.73) |
| Manual | 1.04 (0.85 – 1.29) |
|  |  |
| *Maternal smoking*  No  Yes | Ref  0.81 (0.46 – 1.45) |
| *Breastfed*  No  Yes | Ref  0.06 (0.04 – 0.11) |
|  |  |
| *Kitchen location*  Inside | Ref |
| Separate building | 0.83 (0.70 – 0.99) |
| Outside | 0.73 (0.61- 0.88) |
|  |  |
| *Smoking in the household* |  |
| Never | Ref |
| Daily | 1.16 (0.94 - 1.44) |
| Rarely | 1.11 (0.90 -1.39) |
